# Supplementary material for: A Challenge of COVID—19: Associated Infective Endocarditis with Streptococcus gordonii in a Young Immunocompetent Patient
Source: Medicina (Kaunas). 2021 Nov 26;57(12):1298. doi: 10.3390/medicina57121298 (PMC8707720; doi:10.3390/medicina57121298)
Supplement: Supplementary file 1 [file medicina-57-01298-s001.zip › medicina-1458394-supplementary.pdf]

Table S1. Dynamics of lab data in a patient with COVID-19 infection and infective endocarditis

	Normal Value	Day-1	Day-14	Day-30	Day-60	Day-180
Leucocytes (/mm³)	4000–10000	7100	6300	8200	5100	5200
Neutrophils %	50–62	73.1	62.9	70.9	65.5	60.2
Lymphocytes %	25–40	19	34.7	26	28.8	31.4
Hemoglobin (g/dl)	11,5–16	11.1	10.4	11.8	12.3	12.4
C-reactive protein (mg/L)	0–5	67.12	21.24	4.8	1.94	1.1
ESR (mm/h)	42	92	32	36	16	12
Fibrinogen (mg/dl)	196–372	439	426	629	244	220
Procalcitonin	neg	<0.5	neg	neg	neg	neg
Lactic acid (mg/dl)	4.5–20	5.91	28.4	14.31	10.6	3.8
D-Dimer (ng/ml)	0–500	661.9	536.98	417	360	320
ALT (U/L)	0–34	66	124	429	102	32
HIV		neg				
HBsAg & HBc-Ab		neg		neg		neg
VHC-Ab		neg		neg		
IgM-VHA				neg		
Rheumatoid Factor		neg		neg	neg	neg
Hematuria		+++	++	-	-	-
